# Prognostic Role of Long Noncoding RNAs in Oral Squamous Cell Carcinoma: A Meta-Analysis

**DOI:** 10.1155/2021/6407528

**Published:** 2021-12-26

**Authors:** Yu Wang, Peng Wang, Xin Liu, Ziran Gao, Xianbao Cao, Xilong Zhao

**Affiliations:** ^1^Cell Therapy Technology Transfer Medical Key Laboratory of Yunnan Province, Basic Medical Laboratory, 920th Hospital of Joint Logistics Support Force, PLA, Kunming, 650032 Yunnan Province, China; ^2^Department of Otolaryngology, First People's Hospital of Yunnan Province, Kunming, 650032 Yunnan Province, China; ^3^Faculty of Environmental Science and Engineering, Kunming University of Science and Technology, Kunming, 650032 Yunnan Province, China; ^4^Qujing 69 Hospital, China RongTong Medical Healthcare Group co., Ltd, Qujing, 655000 Yunnan Province, China

## Abstract

Long noncoding RNAs (lncRNAs) have emerged as critical regulators of tumor progression, and lncRNA expression levels could serve as a potential molecular biomarker for the prognosis and diagnosis of some cancers. However, the prognostic value of lncRNAs in oral squamous cell carcinoma (OSCC) remains unclear. Thus, a meta-analysis was conducted to explore the potential prognostic value of lncRNAs in OSCC. We systematically searched PubMed, EBSCO, Web of Science, and Elsevier from 2005 to 2021 to identify all published studies that reported the association between lncRNAs and prognosis in OSCC. Then, we used meta-analytic methods to identify the actual effect size of lncRNAs on cancer prognosis. The hazard ratios (HRs) with 95% confidence intervals (95% CIs) were calculated to assess the strength of the association. The reliability of those results was then examined using measures of heterogeneity and testing for selective reporting biases. According to the inclusion and exclusion criteria, a total of 17 studies were eligible in our meta-analysis, involving 1384 Asian patients. The results identified a statistically significant association of high lncRNA expression with poor overall survival [adjusted pooled hazard ratio (AHR) = 1.52; 95% confidence interval (CI): [1.26–1.84], *p* ≤ 0.001]. The present meta-analysis demonstrated that lncRNA expression might be used as a predictive prognostic biomarker for Asian patients with OSCC.

## 1. Introduction

Oral squamous cell carcinoma (OSCC) is a significant subgroup of head and neck squamous cell carcinomas [1, 2]. OSCC is characterized by invasive growth, frequent metastases, and high recurrence, and its incidence is increasing, with more than 274,000 new patients with OSCC every year worldwide [3–5]. Although considerable developments in diagnosis and combined treatments have been made in recent years, the 5-year survival rate among OSCC patients has not improved and remains less than 50% [6–8]. Therefore, it is essential to identify useful biomarkers and therapeutic targets to improve the prognosis of OSCC.

Long noncoding RNAs (lncRNAs), a class of regulatory transcripts, are synthesized by RNA polymerase II and have lengths greater than 200 nucleotides [8–10]. Recent studies have shown that dysregulated lncRNAs play essential roles in tumor cellular processes of cell proliferation, differentiation, and invasion during cancer development and progression and play essential roles in tumorigenesis and progression of ovarian [11], colorectal [11], gastrointestinal [12], and lung cancers [13]. lncRNAs are regarded as essential therapeutic targets [14, 15].

Some studies have shown that abnormal lncRNAs contribute to biological behaviors, clinical diagnosis, prognosis, and treatment options in OSCC. HOX antisense intergenic RNA (*HOTAIR*) is a transacting lncRNA that was the first identified lncRNA [16]. *HOTAIR* is located at chromosome 12q13.13, which is a regulatory boundary in the HOXC cluster [17]. The expression level of *HOTAIR* was significantly associated with metastasis, tumor differentiation, malignant degree, and prognosis of the patients. In addition, the upregulation of *HOTAIR* expression promoted OSCC cell proliferation, invasion, metastasis, and angiogenesis by binding to *EZH2* and *H3K27me3* and ultimately E-cadherin gene silencing [18]. *H19* acts as an oncogene in OSCC by competing with miR-138 and releasing *EZH2*, thereby playing a role in cell proliferation, migration, invasion, apoptosis, and epithelial-mesenchymal transition (EMT), and high expression of *H19* was correlated with TNM stage, lymph node metastasis, and poor prognosis outcome [19]. One study demonstrated that the low expression of lncRNA AC012456.4 contributed to poor disease-free survival (DFS) and indicated that lncRNA AC012456.4 remarkably correlated with the JNK-STAT and MAPK signaling pathways in tumorigenesis and functioned as a novel target for the diagnosis, clinical treatment, and outcome of OSCC [20]. Due to varying diagnostic accuracy, limitations in sample size, different lncRNA types, and research methods, a single-center study may be inaccurate and inadequate. Based on the current research situation, the present study was aimed at clarifying the clinical feasibility of lncRNAs as potential biomarker candidates by systematically summarizing all eligible articles.

## 2. Materials and Methods

### 2.1. Search Strategy

We conducted this meta-analysis according to the Preferred Reporting Items for Systematic Reviews and Meta-Analyses (PRISMA) guidelines [21]. A systematic literature review was searched from the PubMed, EBSCO, Elsevier, Web of Science, and Elsevier databases for papers online from 2005 to 2021. The search was performed by two independent researchers (YW and XL). The following search terms were used: (oral squamous cell carcinoma or OSCC) and (lncRNA or (long noncoding RNA) or (long noncoding RNAs)) and (prognosis or prognostic or survival). In addition, the cited references in the eligible studies were also searched and reviewed.

### 2.2. Inclusion and Exclusion Criteria

Inclusion criteria are as follows: (1) the research design was a prospective or retrospective study, (2) the paper researched the relationship between lncRNA and the prognosis of survival in OSCC, (3) the hazard ratio and 95% CI of overall survival were reported or could be calculated from the study, (4) more than 20 cases were included, and (5) studies were published in the English language. Two researchers (YW and XL) decided the ultimate eligible studies, and disagreements were resolved by consulting a third researcher (XBC). The exclusion criteria were as follows: (1) studies without sufficient or usable data; (2) reviews, laboratory articles, letters, unpublished data, and conference abstracts; and (3) duplicate publications.

### 2.3. Data Extraction and Quality Evaluation

Two investigators (YW and PW) perused the full text of the included articles and extracted relevant data independently from the eligible studies. Extracted information included the name of the first author, published year, regions, sample size, lncRNA types, HR and 95% CI, case number, outcome, HR estimation, and cutoff value [22]. The Newcastle–Ottawa quality assessment scale (NOS) was used to assess the quality of the included studies [23]. NOS scores of 1–3, 4–6, and 7–9 were designated as low, medium, and high quality, respectively. The quality evaluation was conducted by XL and PW independently, and disagreements were resolved through group discussion with a third investigator (XLZ).

### 2.4. Statistical Methods

*p* < 0.05 was considered statistically significant for comparing the groups with high and low expression of lncRNAs regarding survival of OSCC patients. *p* ≥ 0.05 was identified as no statistically significant difference between the two groups in OSCC patients.

HRs (HRs and 95% CIs) were calculated using a reported method [24] and used to evaluate the overall survival effect. If included articles reported the HR and 95% CI or did not directly provide the HR, but they reported the O-E value (observed value minus expected value), the 95% CI or the log-rank *p* value, we could calculate accurate HR_S_. If only the total number of cases, the number of each group, and the log-rank *p* value were reported, the approximate HRs could be calculated as described previously [24]. Additionally, if only valid data were provided in the form of survival curves, the data from Kaplan–Meier survival curves could be used to calculate HRs by Parmar's method [25].

Statistical heterogeneity within studies was detected by the *Q* statistic and *I*^2^ statistics. If *I*^2^ ≤ 50% identified lower heterogeneity, a fixed-effect model was used. If *I*^2^ > 50% showed higher heterogeneity, the random-effect model was used [26]. Subsequently, Egger's method was used to detect publication bias and observed in the form of a funnel plot [27]. If publication bias was found, then the HRs were adjusted by the method of Duval and Tweedie's trim-and-fill [28].

## 3. Results

### 3.1. Literature Search and Characteristics of the Included Studies

**Figure 1:**
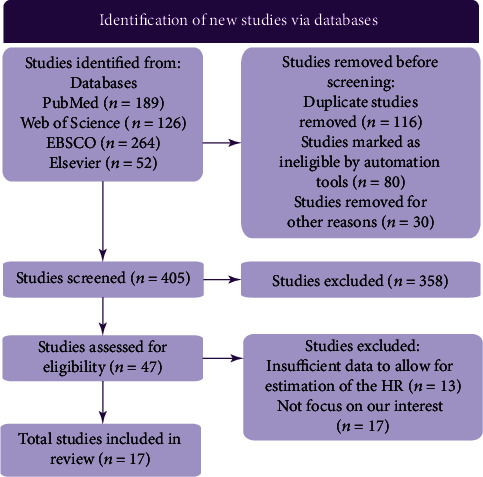
A flowchart of the article search.

As shown in Figure 1, 631 articles were searched in the databases of PubMed, Web of Science, EBSCO, and Elsevier. After removing duplicate studies and ineligible studies, 405 studies remained. After reading the title, abstract, and keywords and further excluding irrelevant studies (*n* = 358), 47 eligible articles were downloaded and analyzed in detail. Seventeen articles were excluded because HR could not be calculated, and 13 articles were excluded because they did not focus on our area of interest. In the end, 17 studies were included in this review [19, 20, 29–43]. The necessary information and data from the included studies are shown in Tables 1 and 2. The studies enrolled 1384 participants, with a maximum sample size of 252 and a minimum sample size of 30 patients. Eligible studies published from 2013 to 2021 reported an association between lncRNA expression level and overall survival, and all participants' ethnic backgrounds were Asian. In addition, lncRNAs and relevant targets in oral squamous cell carcinoma are shown in Table 3.

### 3.2. Quality Evaluation

The data were extracted from all 17 eligible studies. According to the NOS quality assessment system, 9 studies were of high quality, 6 studies were of medium quality, and 2 studies were of low quality (Table 1). The average score of all included studies was 6.53. In addition, four studies were based on multivariate analysis, and 13 studies were based on univariate analysis. HR and 95% CI of each study are shown in Table 2.

### 3.3. Meta-Analysis

**Figure 2:**
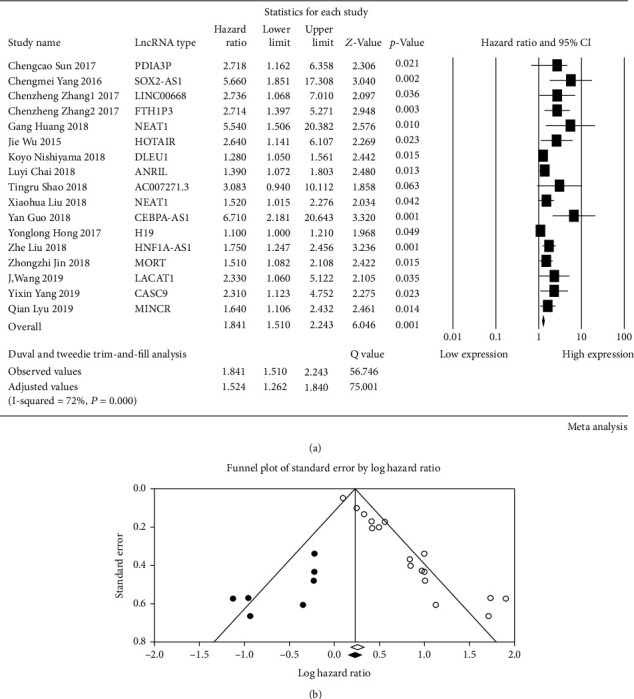
A meta-analysis evaluating the hazard ratio of lncRNA expression and the overall survival of OSCC. (a) The forest plot for evaluating all included studies. *I*^2^ = 71.80% was identified as higher heterogeneity, and the random-effect model was used. Publication bias was found, and HRs were adjusted by Duval and Tweedie's trim-and-fill method. (b) The funnel plot for detecting publication bias. Observed studies were represented by white circles. Possibly missed studies, represented by black circles, were imputed by Duval and Tweedie's trim-and-fill method. The observed and theoretical combined effect sizes were represented by white and black rhombuses, respectively.

The meta-analysis data of pooled HRs of overall survival were extracted from the 17 included studies. The results showed a pooled HR of 1.52 (95% CI, 1.26–1.84; *p* < 0.001) with statistically significant heterogeneity (*Q*-statistic, 75.00; *I*^2^ = 71.80%, *p* value < 0.001, random-effect model) (Figures 2(a) and 2(b)). Compared with the decreased lncRNA expression group, upregulated lncRNA expression was correlated with poor prognosis.

**Figure 3:**
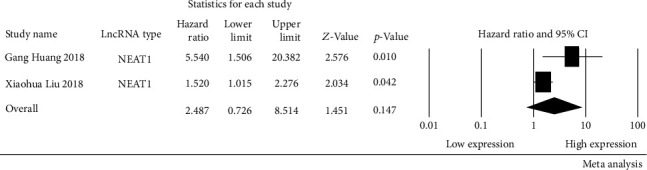
Forrest plots of studies evaluating hazard ratios of NEAT1 and the overall survival of OSCC patients. *I*^2^ = 71.05% was identified as higher heterogeneity, and the random-effect model was used.

Most of the lncRNAs were investigated in a single study; only *NEAT1* was investigated in two studies. We then conducted a meta-analysis to assess the relationship between *NEAT1* expression and overall survival (OS) in OSCC patients. We noted that the heterogeneity was significant (*I*^2^ = 71.05%, *p* = 0.06). Therefore, a random-effect model was applied, and the results of the analysis showed that *NEAT1* was not significantly associated with OS (HR: 2.49, 95% CI: 0.73-8.51; *p* = 0.15) (Figure 3).

Subsequently, we conducted subgroup analyses according to univariate and multivariate analyses, NOS score evaluation, and source of HR. The results are shown in Table 4. The combined analysis showed that upregulated lncRNA expression has significant prognostic value in OSCC: univariate analysis (AHR: 1.43, 95% CI: 1.20–1.71, *p* < 0.001), multivariate analysis (AHR: 2.50, 95% CI: 1.65–3.78, *p* < 0.001), source of HR (reported: HR: 1.85, 95% CI: 1.51-2.26, *p* < 0.001; survival curve: AHR: 1.18, 95% CI: 1.10-1.27, *p* < 0.001), and NOS score evaluation (high: 1.64, 95% CI: 1.38–1.96, *p* < 0.001; medium: 1.45, 95% CI: 1.01–2.07, *p* = 0.04; low: 3.78, 95% CI: 1.92-7.44, *p* < 0.001).

Publication bias of the included articles was evaluated by funnel plots and Begg's bias test. The shape of the funnel plot was asymmetrical, and the *p* value of Begg's test was 0.002 for OS of all enrolled articles, suggesting the existence of significant publication bias in the meta-analysis. Then, we use Duval and Tweedie's trim-and-fill method to adjust the HRs. The outcome of this study was adjusted for HRs.

## 4. Discussion

Oral squamous cell carcinomas (OSCC) are often detected at an advanced clinical stage with metastasis, and poor prognosis of oral cancer may lead to high incidence [44]. Despite considerable advances being achieved in medical technologies for cancer diagnosis and treatment in the past decades, the 5-year survival rate for patients with OSCC remains less than 50% [45].

Accumulating evidence reveals that lncRNAs serve critical regulatory roles in diverse biological processes, including gene expression, cell invasion, migration, and tumorigenesis [46]. Previous meta-analyses have demonstrated high expression of lncRNAs to correlate with poor prognosis in patients with various cancers, such as ovarian [11], colorectal [11], gastrointestinal [12], and lung cancers [13]. However, no meta-analyses have revealed the role of lncRNAs in OSCC prognosis.

We conducted a meta-analysis to validate the accuracy and value of the theoretical results of lncRNAs as prognostic molecular markers in patients with OSCC. A total of 17 studies, including 1384 patients, were enrolled within our meta-analysis. The expression of *PDIA3P*, *SOX21-AS1*, *LINC00668*, *FTH1P3*, *NEAT1*, *HOTAIR*, *DLEU1*, *ANPIL*, *CEBPA-AS1*, *H19*, *HNF1AAS1*, *MORT*, *LACAT1*, *CASC9*, and *MINCR* was upregulated. There are no downexpression lncRNAs in participants of this analysis. The analysis showed a reliable result for upregulated lncRNA expression to correlate with poor prognosis in OSCC (HR: 1.52, 95% CI: [1.26, 1.84]; *p* < 0.001, random effect). Also, subgroup analysis revealed that lncRNA expression correlated with prognosis, while the analysis method, source of HR, and NOS score evaluation did not significantly affect the pooled results of this meta-analysis. By our analysis, these findings suggest that lncRNA can be developed as a prognostic and therapeutic biomarker in OSCC.

Several lncRNAs with high HR in this study have also been reported in other cancers other than OSCC accidents. For example, *CEBPA-AS1* with high HRs (HR: 6.71, 95% CI: 3.61-8.73) was also reported in gastric cancer. Ke et al. found that high expression of *CEBPA-AS1* has a poor prognosis patients with gastric cancer [47]. *HOTAIR*, as one of the most crucial lncRNA, has been extensively studied, and overexpression *HOTAIR* is correlated with poor survival for breast, colon, and liver cancer patients [48]. This study also showed that patients with high expression *HOTAIR* have a poor prognosis in OSCC. While the prognostic value of *NEAT1* was assessed, and the pooled HRs were 2.49 (95% CI: 0.73–8.51, *p* = 0.15, random effect), the results showed that *NEAT1* was not statistically significantly associated with OS. Only two studies were included in this evaluation, resulting in a low power of evidence. *NEAT1* has been found to be associated with many different types of cancer prognosis. Fu et al. identified that lncRNA *NEAT1* was overexpressed in gastric cancer tissues and cell lines, and patients with high levels of *NEAT1* had more reduced survival than those with lower levels of *NEAT1* [49]. Chen et al. found that high expression of *NEAT1* predicts poor prognosis and has a crucial regulatory role in esophageal squamous cell carcinoma [50]. Therefore, further research needs to confirm the mechanisms of *NEAT1* in the progression of OSCC.

In this study, we also collected mechanisms of lncRNAs; 9 of the included studies investigated the correlation between lncRNAs and microRNAs. It could be evidenced from these researches that the relationship between lncRNAs and microRNAs is associated with cancer incidence. The same lncRNA is associated with the occurrence and development of different cancers, but the mechanism is still unclear. In the future, research hotspots may be focused on the method of simultaneous intervention with multiple RNA by exploring the interrelationship between lncRNA and multiple types of RNA.

It should be stressed that there are several limitations in our meta-analysis. Firstly, only 17 studies were eligible in this meta-analysis, which might weaken the reliability of our results. Secondly, remarkable statistical heterogeneity (*I*^2^ = 71.80%) was observed, which may be due to the differences in cancer types, internal control, cutoff value, clinical characteristics, and sample sizes. The geographical bias may be present, as all studies were performed in Asia. As demonstrated in previous studies, people of different race/ethnicity vary in their risk of developing OCSS, and the differences in OS may link both to the genetic and the lifestyle. We hope that other countries in different regions will also conduct relevant research and reports in the future. Finally, some HRs were extracted from the survival curves, which may lead to small statistical errors.

## 5. Conclusion

Despite several limitations described above, the meta-analysis offers evidence that upregulated lncRNAs are significantly corrected with poor OS in Asian patients with OSCC, which demonstrated that the lncRNAs could serve as the prognostic factor for Asian patients with OSCC. However, large-scale and comprehensive studies are needed to improve the credibility of our findings and thus promote the clinical utility of lncRNAs in OSCC prognosis evaluation.

## Figures and Tables

**Table 1:** Necessary information about the included studies.

Study ID	lncRNA	Country	Sample	Reference	Detection method	Sample size	Outcome	Source of HR	Cutoff value	NOS
Jie Wu, 2015 [18]	HOTAIR	China	Tissues	GAPDH	qPCR	100	OS	Log rank	Median	7
Yonglong Hong, 2017 [19]	H19	China	Tissues	GAPDH	qPCR	42	OS	Sur curve	NA	6
Luyi Chai, 2018 [29]	ANRIL	China	Tissues	GAPDH	qPCR	130	OS	Sur curve	Median	6
Yan Guo, 2018 [30]	CEBPA-AS1	China	Tissues	GAPDH	qPCR	60	OS	Reported	Median	3
Gang Huang, 2018 [31]	NEAT1	China	Tissues	NEAT1/RGS20	qPCR	30	OS	Sur curve	NA	8
Xiaohua Liu, 2018 [32]	NEAT1	China	Tissues	GAPDH	qPCR	58	OS	Sur curve	Median	7
Koyo Nishiyama, 2018 [33]	DLEU1	Japan	Tissues	ACTB (*β*-actin)	qPCR	252	OS	Sur curve	Median	8
Tingru Shao, 2018 [34]	AC0077271.3	China	Tissues	GAPDH	qPCR	80	OS	Reported	Median	6
Chengcao Sun, 2017 [35]	PDIA3P	China	Tissues	GAPDH	qPCR	58	OS	Sur curve	NA	3
Chengmei Yang, 2016 [36]	SOX21-AS1	China	Tissues	GAPDH	qPCR	86	OS	Reported	Median	6
Chenzheng Zhang2, 2017 [37]	FTH1P3	China	Tissues	GAPDH/U6	qPCR	70	OS	Log rank	Mean	9
Chenzheng Zhang1, 2017 [38]	LINC00668	China	Tissues	GAPDH/U6	qPCR	50	OS	Log rank	Mean	8
Zhongzhi Jin, 2018 [39]	MORT	China	Tissues	GAPDH	qPCR	59	OS	Reported	Median	7
Zhe Liu, 2018 [40]	HNF1A-AS1	China	Tissues	GAPDH	qPCR	62	OS	Sur curve	Median	5
Qian Lyu, 2019 [41]	MINCR	China	Tissues	GAPDH	qPCR	80	OS	Sur curve	Median	6
J, Wang, 2019 [42]	LACAT1	China	Tissues	GAPDH	qPCR	78	OS	Sur curve	Median	7
Yixin Yang, 2019 [43]	CASC9	China	Tissues	GAPDH	qPCR	84	OS	Reported	Median	9

**Table 2:** Characteristics of the included studies.

lncRNAs	Reference	U&M analysis	Case number		OS	
			High expression	Low expression	HR (95% CI)	*p* value
HOTAIR	Jie Wu, 2015 [18]	U	30	70	2.64 (1.14-6.10)	0.02
H19	Yonglong Hong, 2017 [19]	U	25	17	1.10 (1.0-1.21)	0.05
ANRIL	Luyi Chai, 2018 [29]	U	57	73	1.39 (1.07-1.80)	0.01
CEBPA-AS1	Yan Guo, 2018 [30]	U	30	30	6.71 (3.61-8.73)	<0.001
NEAT1	Gang Huang, 2018 [31]	M	12	18	5.54 (1.5120.38)	0.01
NEAT1	Xiaohua Liu, 2018 [32]	U	26	32	1.52 (1.02-2.28)	0.04
DLEU1	Koyo Nishiyama, 2018 [33]	M	126	126	1.28 (1.05-1.56)	0.01
AC0077271.3	Tingru Shao, 2018 [34]	U	40	40	3.08 (0.95-10.02)	0.06
PDIA3P	Chengcao Sun, 2017 [35]	U	32	26	2.72 (1.62-6.36)	<0.001
ANRIL	Chengmei Yang, 2016 [36]	U	57	73	1.39 (1.07-1.80)	0.01
SOX21-AS1	Chenzheng Zhang2, 2017 [37]	M	57	29	5.66 (1.85-17.30)	0.002
FTH1P3	Chenzheng Zhang1, 2017 [38]	U	37	33	2.71 (1.40-5.27)	0.003
LINC00668	Zhongzhi Jin, 2018 [39]	U	15	35	2.74 (1.07-7.01)	0.03
MORT	Zhe Liu, 2018 [40]	U	31	28	1.51 (1.08-2.11)	0.02
HNF1A-AS1	Qian Lyu, 2019 [41]	U	32	30	1.75 (1.25-2.46)	<0.001
MINCR	J, Wang, 2019 [42]	U	40	40	1.64 (1.11-2.43)	0.01
LACAT1	Yixin Yang, 2019 [43]	U	34	44	2.33 (1.06-5.12)	0.04
CASC9	Jie Wu, 2015 [18]	M	53	31	2.31 (1.12-4.75)	0.02

**Table 3:** lncRNAs and relevant targets in oral squamous cell carcinoma.

lncRNAs	Poor prognosis	Role	Relevant targets	Function	Reference
HOTAIR	Upregulated	Oncogene	Cyclin D1, EGFR， c-Myc	Proliferation/invasion/metastasis/angiogenesis	[18]
H19	Upregulated	Oncogene	miR-138, EZH2	Proliferation/invasion/apoptosis/EMT	[19]
ARNIL	Upregulated	Oncogene	miR-125a, ESRRA	Proliferation/invasion/migration	[29]
CEBPA-AS1	Upregulated	Oncogene	CEBPA, Bcl2	Proliferation/invasion/migration/apoptosis	[30]
NEAT1	Upregulated	Oncogene	miR-365, RGS20	Migration/invasion/progression	[31, 32]
DLEU1	Upregulated	Oncogene	HA-CD44	Proliferation/invasion/migration	[33]
AC007271.3	Upregulated	Oncogene	*β*-Catenin, CyclinD1, c-muc, Bal-2	Proliferation/invasion/migration	[34]
PDIA3P	Upregulated	Oncogene	miR-185-5p, CCND2	Proliferation.	[35]
SOX21-AS1	Upregulated	Oncogene	miR-145	Proliferation/invasion/growth	[36]
FTH1P3	Upregulated	Oncogene	miR-224-5p	Proliferation	[37]
LINC00668	Upregulated	Oncogene	miR-297/VEGFA	Progression	[38]
MORT	Upregulated	Oncogene	ROCK1	Proliferation	[39]
HNF1A-AS1	Upregulated	Oncogene	STAT3	Proliferation/migration/EMT	[40]
MINCR	Upregulated	Oncogene	Wnt/*β*-catenin	Proliferation/invasion	[41]
LACAT1	Upregulated	Oncogene	MicroRNA-4301	Proliferation/differentiation	[42]
CASC9	Upregulated	Oncogene	AKT/mTOR	Proliferation/apoptosis/autophagy/progression	[43]

**Table 4:** Subgroup analyses of the prognosis of OSCC patients with lncRNA expression.

Studies (*n*)	HR (95% CI)	*p* value	Heterogeneity *I*^2^ (%)	*p* value	*p* for Begg (2-tailed)	*p* for Egger (2-tailed)	Pub. bias	AHR^a^ (95% CI)	*p* value
All studies (17)	1.84 (1.51-2.24)	<0.001	71.80	<0.01	<0.01	<0.01	Yes	1.52 (1.26-1.84)	<0.001
U&M analysis									
Univariate (13)	1.62 (1.35-1.95)	<0.001	67.48	<0.01	<0.01	<0.01	Yes	1.43 (1.20-1.71)	<0.001
Multivariate (4)	3.51 (2.16-5.71)	<0.001	9.57	0.35	0.50	0.22	Yes	2.50 (1.65-3.78)	<0.001
Source of HR									
Sur curve (9)	1.45 (1.20-1.74)	<0.001	66.98	<0.01	<0.01	<0.01	Yes	1.18 (1.10-1.27)	<0.001
Reported (8)	2.19 (1.74-2.74)	<0.001	37.28	0.12	0.04	<0.01	Yes	1.85 (1.51-2.26)	<0.001
NOS score^b^									
High (9)	1.91 (1.56-2.32)	<0.001	11.96	0.34	0.04	<0.01	Yes	1.64 (1.38-1.96)	<0.001
Medium (6)	1.92 (1.33-2.78)	<0.001	79.36	<0.01	0.05	<0.01	Yes	1.45 (1.01-2.07)	0.04
Low (2)	3.78 (1.92-7.44)	<0.001	36.72	0.21	—	—	NO	—	—

Abbreviations: HR: hazard ratio; CI: confidence interval; Pub. bias: publication bias; AHR: adjusted HR; U&M analysis: univariate & multivariate analysis. ^a^AHR: if publication bias was found, the HRs were adjusted and reevaluated; if the number of combined studies was not >3, the publication bias could not be analyzed. ^b^NOS score: the NOS score was used to evaluate the quality of the included studies, and NOS scores of 1–3, 4–6, and 7–9 were considered to indicate low, medium, and high quality, respectively.

## Data Availability

The data used to support the findings of this study are included within the article.
